# Resolving Single-Particle Absorption and Scattering by Plasmonic Magnesium Nanoparticles

**DOI:** 10.1021/acs.nanolett.6c00484

**Published:** 2026-04-01

**Authors:** Claire A. West, Tinglian Yuan, Tathagata Chatterjee, Vladimir Lomonosov, Jae-Ho Kim, Emilie Ringe, Stephan Link

**Affiliations:** † Department of Chemistry, Department of Electrical and Computer Engineering, Materials Research Lab, 14589 University of Illinois at Urbana−Champaign, Urbana, Illinois 61801, United States; ‡ Department of Earth Sciences, 2152 University of Cambridge, Downing Street, Cambridge CB2 3EQ, United Kingdom; § Department of Materials Science and Metallurgy, University of Cambridge, 27 Charles Babbage Road, Cambridge CB3 0FS, United Kingdom; ○ Department of Chemistry, 14589 University of Illinois at Urbana−Champaign, Urbana, Illinois 61801, United States

**Keywords:** alternative plasmonic materials, colloids, single-particle spectroscopy, photothermal imaging, dark-field scattering

## Abstract

Magnesium nanoparticles have emerged as a promising plasmonic material due to their low cost and biocompatibility, yet their optical absorption at the single-particle level is largely uncharacterized. While ensemble extinction measurements of 170 nm Mg spheroids show a broad extinction spectrum, we demonstrate through correlated single-particle dark-field scattering and photothermal absorption spectroscopies that individual nanoparticles support well-defined plasmon resonances in absorption and scattering. We found that the peaks in absorption and scattering occur at a similar wavelength average, with absorption consistently broader than scattering. Simulations reproduce these trends and confirm that the broader absorption line width arises from the large dispersion of the real part of Mg’s dielectric function. These findings provide fundamental insights into the spectral differences in absorption and scattering by Mg nanoparticles and demonstrate the necessity of single-particle measurements for understanding their optical response, crucial for optimizing performance in diverse plasmonically powered applications.

## Graphical abstract

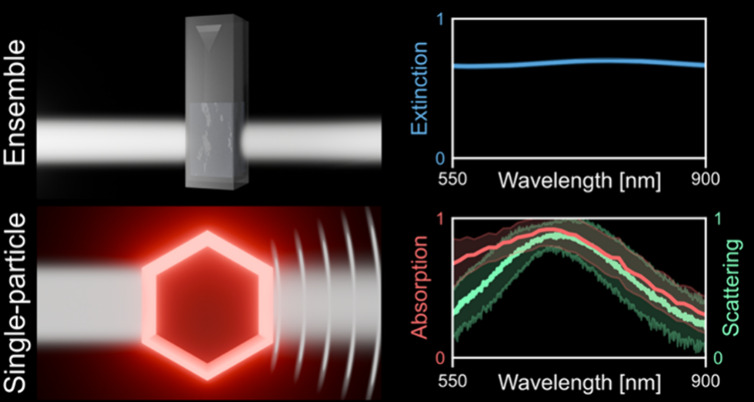

Novel metallic nanoparticles (NPs) and architectures are continuously being developed to meet the needs of plasmonics-driven applications including photocatalysis,^1,2^ surface enhanced spectroscopies such as surface-enhanced Raman spectroscopy (SERS),^3,4^ refractive index sensing,^5^ and photothermal therapy.^6^ Current efforts in this arena include using alternative metals,^7–9^ creating exotic NP geometries,^10,11^ constructing two or three-dimensional composite structures,^12–14^ synthesizing homogeneous and heterogeneous alloys,^15,16^ and designing hybrid plasmonic NPs with other metal and nonmetal components.^17^ This research into plasmonic NP design is driven by the need to optimize the physical and chemical properties of NPs for each specific application.^18^ For example, plasmon-assisted photocatalysis requires efficient light absorption and concentration across the solar spectrum, chemical stability, and tailored selectivity for specific target molecules. SERS requires intense local electric fields or “hot spots” where analyte molecules can easily adsorb, chemical stability, and for, biomedical sensing, negligible heat generation and low cytotoxicity. Lastly, photothermal therapy requires light absorption where biological media is most transparent (“biological window” at approximately 650–1350 nm), efficient light-to-heat conversion, and short- and long-term biocompatibility. As such, developing novel plasmonic NPs and characterizing their optical response is crucial to creating materials that meet these application-specific design requirements.

Mg, in the nonoxidized, metallic form, emerged as a promising new plasmonic material^19–22^ because it is less expensive and more biocompatible^23^ compared to other plasmonic metals, including Ag, Au, Al, and Cu. While Mg’s plasmonic character was initially observed in nanofabricated nanodisk arrays,^24^ synthesis protocols resulting in stable, metallic colloidal NPs that support tunable plasmon resonances have been recently reported.^25–30^ Mg NPs form a thin, approximately 10 nm self-limiting oxide shell on the surface and are stable in organic solvents at room temperature, and in air at temperatures up to 400 °C.^25,31–35^ Synthesis conditions can be tuned to produce either primarily single-crystalline hexagonal platelets and singly twinned NPs,^36^ or faceted spheroidal NPs.^26,28^ As synthesized, Mg NPs are stable in ambient conditions and in oxygen-rich environments,^32^ but react in the presence of water.^37^ Mg NPs can be decorated with different metals via galvanic replacement^38^ and stabilized in aqueous media with various coatings.^37^ Mg NPs have been proven to function as SERS substrates,^35^ light antenna for plasmon-assisted catalysis,^39,40^ and produce sufficient heat for photothermal therapy applications.^41^ These studies demonstrate that Mg NPs support tunable plasmonic properties, yet single-particle characterizations are required to further guide NP design and optimization for diverse applications.

Single-particle spectroscopies enable characterization of the size- and morphology-dependent optical properties of NPs and avert averaging effects from ensemble measurements.^42–46^ Dark-field scattering (DFS) and scanning transmission electron microscopy electron energy loss spectroscopy (STEM-EELS) are two approaches useful for probing the optical response of plasmonic NPs at the single-particle level.^47,48^ DFS^25,33,36^ and STEM-EELS^33–36^ of Mg NPs demonstrate the effect of NP heterogeneity on their plasmonic character, i.e., what modes the NPs support, their spectral position, and line width. However, neither approach measures absorption. Characterizing absorption is critical because many applications depend primarily on absorption rather than scattering: most plasmon-assisted catalysis^49–51^ and all thermally mediated plasmonics applications^52–54^ utilize on NP absorption, while refractive index sensing^55,56^ depends on NP scattering and SERS^57^ depends on the NP’s local electric fields. Measuring absorption is significantly more challenging compared to other single-particle techniques. This difference arises from the fact that the direct absorption by a single particle is weak compared to the incident light, while scattering strongly contributes to the overall extinction as well especially when the NPs have diameters approximately greater than 75 nm,^58–61^ and scattered light can furthermore be easily isolated by angled illumination.

While single-particle scattering of plasmonic NPs is easier to measure, the line shape may not always be identical to absorption. Differences between absorption and scattering emerge depending on NP size, material, shape, and environment. Regarding NP size, the largest differences that occur are in the magnitude of each cross-section: small NPs primarily absorb, large NPs primarily scatter.^58,62^ Furthermore, larger NPs can introduce differences in the line shape itself. For example, Ag nanospheres have equivalent peak positions and line widths for diameters less than 50 nm, but with increasing size the scattering peak redshifts and absorption broadens.^63^ With respect to NP material, the most significant differences arise from the presence of interband transitions. For example, the interband transitions manifest themselves as the dominant signal in absorption in Au NPs from the green through the ultraviolet spectral regions, but are absent in scattering.^64,65^ In addition, absorption and scattering peak positions and line widths differ slightly even for small Au nanospheres and nanorods.^62,66–70^ Lastly, both NP shape^71^ and local environment^72,73^ can introduce differences in the overall magnitude, peak positions, and line widths. Thus, to completely characterize the optical response of plasmonic nanomaterials, it is critical to measure and model both absorption and scattering. While single-particle scattering of Mg NPs has been reported before,^25,33,36^ absorption has not. Therefore, a single-particle analysis of the absorption and scattering of Mg NPs is necessary to resolve the photothermal response of individual NPs and determine how heterogeneities impact light absorption.

In this paper, we measure the correlated absorption and scattering of individual faceted spheroidal Mg NPs. Bulk extinction from ensemble ultraviolet–visible–near-infrared (UV–vis–NIR) spectroscopy measurements reveal a broad spectrum. However, correlated measurements of single NPs expose well-defined peaks in both absorption and scattering. Further analysis uncovers that the absorption line widths are larger on average than the scattering line widths. Companion simulations confirm that this trend is a consequence of the optical properties of Mg NPs and specifically the dispersion in Mg’s real part of the dielectric function. Ultimately, single-particle experiments and simulations of Mg NP absorption and scattering offer direct insight into their fundamental plasmonic properties in a way that aids translation of NP design into practical applications.

## Results and Discussion

The faceted spheroidal Mg NPs are synthesized using a seed-mediated growth approach,^28^ with more details provided in Section S1.1. Transmission electron micrographs (TEM) of the NPs (Figure 1A) indicate that the NPs are predominantly spheroidal with hexagonal symmetry and some shape variation. Additional TEM images at different magnifications are included in Figure S1, as well as tilt series micrographs confirming their three-dimensional shape in Figure S2. The mean and standard deviation of the maximum projected lengths of 297 NPs are 168 ± 17 nm (Figure 1B). Ensemble extinction spectroscopy of a colloidal solution containing the Mg NPs suspended in isopropanol reveals a broad spectrum (Figure 1C), with more information provided in Figure S3.

**Figure 1:**
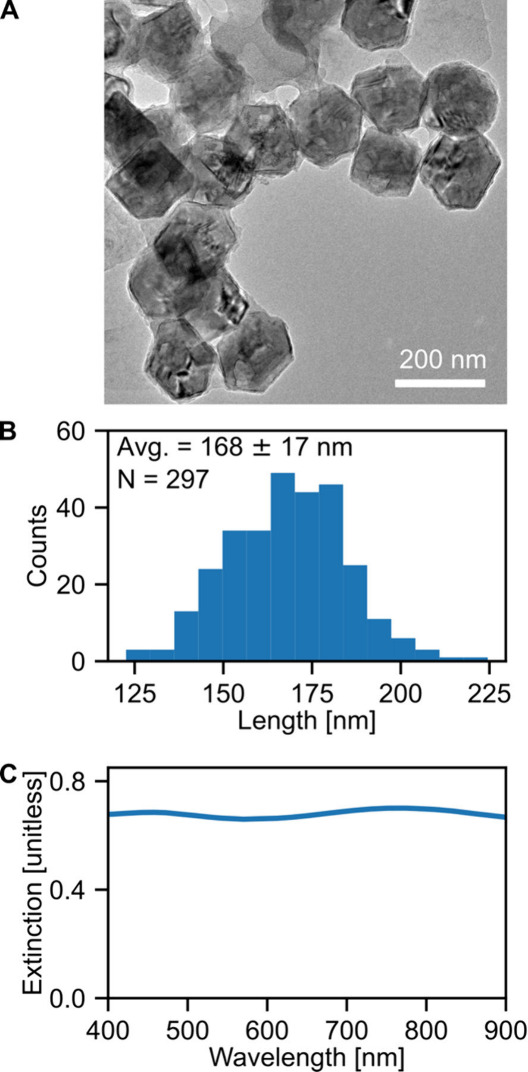
Ensemble characterization of colloidal Mg NPs, indicating small size and shape heterogeneity. (A) Representative TEM image of Mg NPs illustrates the narrow distribution of shapes within a single Mg NP synthesis. (B) Histogram of maximum projected length of 297 Mg NPs with an average NP size of 168 ± 17 nm. (C) Bulk UV–vis–NIR spectrum of colloidal Mg NPs, averaged over repeated measurements shows a broad extinction across visible wavelengths.

Despite the broad extinction spectrum, the Mg NPs are nevertheless expected to support discernible localized surface plasmon resonances because the bulk UV–vis–NIR measurement detects the ensemble extinction produced by the entire colloid. Distinct peaks could be hidden for the following three reasons. First, extinction contains contributions from both the light scattered and absorbed by the NPs. If both processes occur at equal magnitudes yet different peak wavelengths, the measured spectrum would be broadened. However, this assumption of equal absorption and scattering is unlikely the cause because in general nanospheres larger than 150 nm scatter significantly more than they absorb. Second, the peak position and line width of Mg NPs are highly sensitive to exact NP shape and size. Even minor sample heterogeneity could broaden the extinction spectrum and obfuscate plasmon resonances.^25^ Lastly, if NPs aggregate into dimers, trimers, or large multimers, these structures produce large scattering cross sections with a broad line width. As such, even a small percentage of NP aggregation could obscure the plasmon resonances of isolated NPs. Therefore, it is necessary to correlate single-particle spectroscopies with electron microscopy to identify the true optical response of individual Mg NPs hidden by the ensemble extinction.

Correlated single-particle experiments combine DFS and photothermal absorption spectroscopy (PTA)^74–76^ to measure light scattered and absorbed, respectively, with scanning electron microscopy (SEM) to measure the NP size and shape. The NPs are measured in glycerol on a glass coverslip (Figure 2A). DFS is carried out by illuminating the sample with a light cone, such that the incident light that excites the NPs is not collected by the objective (Figure 2B). Instead, only the light scattered by the NPs is collected. We perform PTA spectroscopy by using an amplitude-modulated wavelength-tunable pump laser focused onto an individual NP (Figure 2C). The light absorbed by the NP is converted into heat via nonradiative relaxation and locally changes the refractive index of the surrounding glycerol. A 532 nm probe laser transmits through this glycerol “thermal lens” and its perturbed amplitude is isolated via lock-in detection as the photothermal signal. Because photothermal conversion is the dominant process after light absorption, the photothermal signals as a function of pump wavelength are attributed to the absorption spectrum. DFS and PTA collected here are unpolarized. SEM is then used to determine the size and shape of each measured NP, allowing us to correlate scattering, absorption, and geometry. For more details regarding sample preparation, DFS, and PTA, see Sections S1.2–S1.4 and Figures S4–S6.

**Figure 2:**
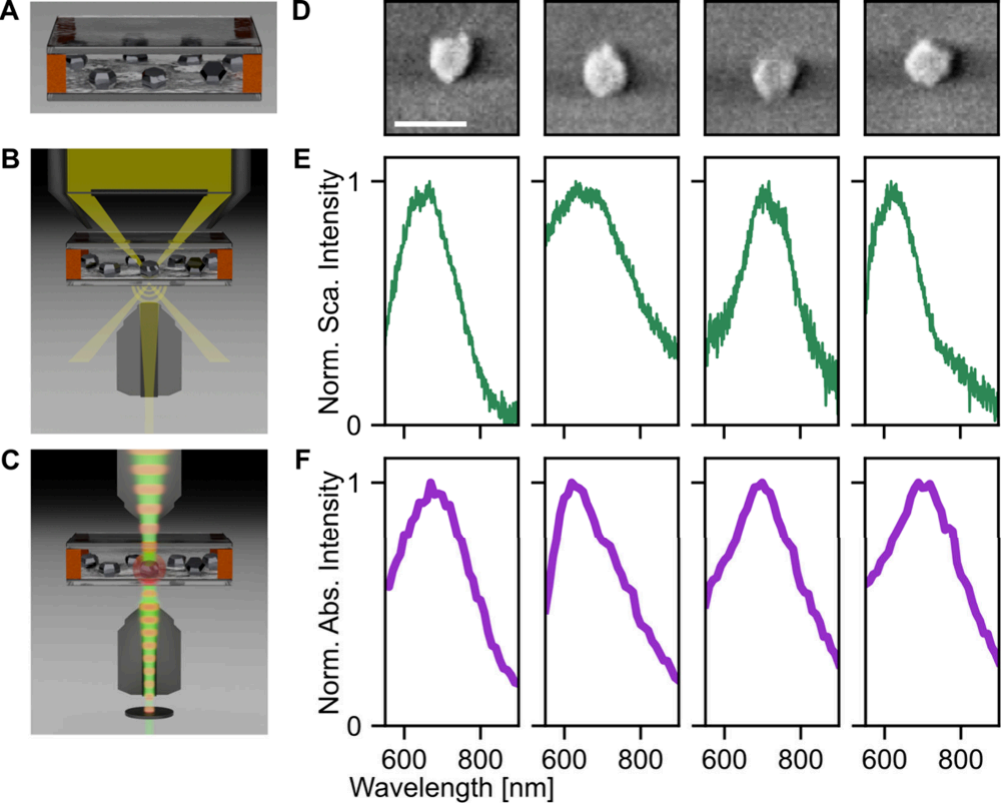
Correlated single-particle scattering and absorption spectra of Mg NPs reveal resonances in the visible spectral range. (A) Schematic of sample geometry. Diagrams of the (B) DFS and (C) PTA experiments, with the modulated red beam corresponding to the pump and the green beam representing the probe. (D) SEM images of four different Mg NPs. The scale bar is 245 nm and applies across all panels. Corresponding (E) normalized scattering and (F) normalized absorption spectra of the NPs from part D. The peak wavelengths in absorption and scattering shift with changing NP morphology and are not identical between absorption and scattering for the same NP.

Measurements on single Mg NPs show well-defined plasmon resonances in the visible wavelength range, with distinct peaks in both absorption and scattering. Four representative NP SEM images and spectra (Figure 2D–F) illustrate that the line shapes for absorption and scattering both vary with small changes in the NP geometry, as known for NPs made from other plasmonic metals.^77,78^ Furthermore, the scattering peak does not necessarily occur at the same wavelength as the corresponding maximum in absorption. Scattering peaks are found at 667, 630, 717, and 618 nm (left to right in Figure 2E), while absorption peaks are found at 670, 620, 700, and 690 nm (left to right in Figure 2F). Furthermore, the full-width at half-maximum (fwhm) among these Mg NPs differs between absorption and scattering: 205, 379, 231, and 145 nm for scattering and 260, 340, 260, and 280 nm for absorption. Given the apparent diversity in the optical response of this subset of Mg NPs, we continue next with an analysis of additional Mg NPs to establish overall trends.

A total of 19 measured single Mg NPs are summarized in Figure 3, with individual spectra provided in Figure S6. First, we create a subensemble scattering spectrum by averaging all scattering measurements (Figure 3A, solid green trace), revealing a peak centered at 690 nm with a fwhm of 210 nm. The variation in peak positions across all measured Mg NPs, first illustrated for 4 NPs in Figure 2E, is visualized by the green shaded area that indicates the corresponding standard deviation in spectral line shape. While this average scattering spectrum is broad, it cannot entirely account for the ensemble UV–vis–NIR spectrum; likely the broadness of the ensemble spectrum is instead due to a small amount of aggregates. The same analysis is done for the single-particle absorption spectra (Figure 3B; the solid purple trace is the average, and the shaded purple trace represents the standard deviation). The average absorption maximum is 690 nm, approximately the same value as for scattering. The fwhm of the average absorption spectrum is 90 nm greater than the average scattering fwhm (300 nm). To investigate these trends further, we return to a correlated single-particle analysis.

**Figure 3:**
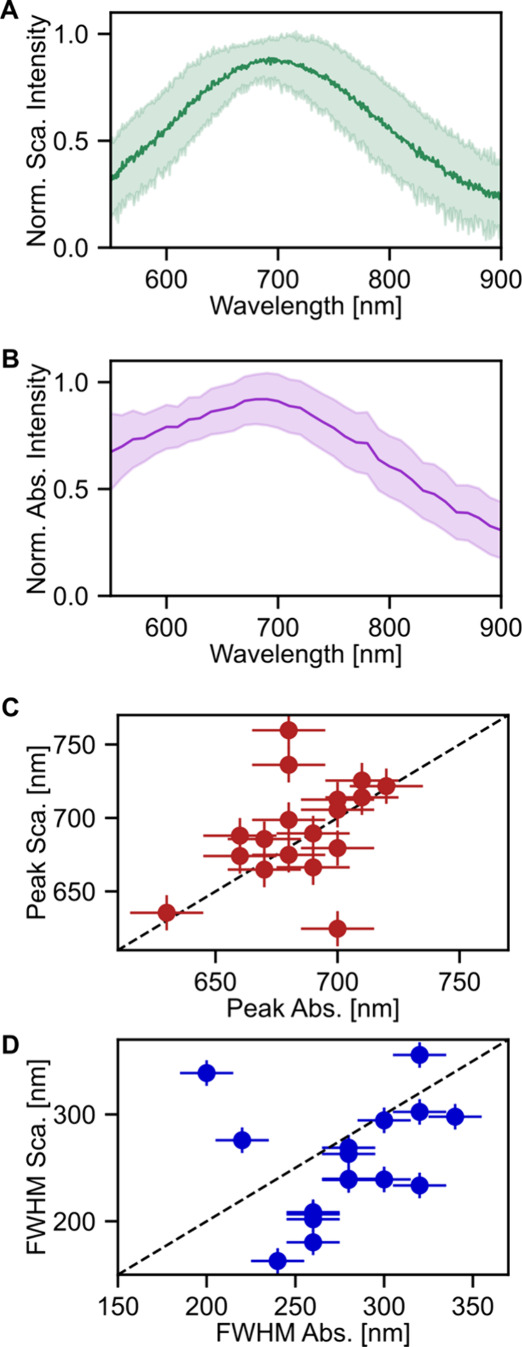
Correlated measurements across 19 Mg NPs reveal that absorption and scattering peaks occur at similar wavelengths but with different line widths. (A) Average scattering (dark green) and standard deviation (shaded green) among all measured single NPs. (B) Average absorption (dark purple) and standard deviation (shaded purple) among all measured single Mg NPs. (C) Correlation of extracted peak absorption position against peak scattering position. The dashed line indicates a one-to-one correlation. On average, the absorption and scattering peak wavelengths are directly correlated. (D) Same as part C, except for the fwhm values. The absorption spectra are on average broader than the scattering spectra.

The absorption and scattering peak wavelength positions of the 19 Mg NPs follow each other at the individual NP level (Figure 3C). To determine the peak wavelength, the data are denoised with Gaussian blur, and the maximum value of the spectrum is extracted. Each marker in Figure 3C represents data from a single NP. The spectra do not fit well to Lorentzian or Gaussian functions, as expected for large plasmonic NPs supporting multiple modes. The error bars for each data point reflect the kernel of the Gaussian blur. The dashed line is a one-to-one correlation between the absorption and scattering peak wavelengths. For most NPs, there is approximately a one-to-one correlation between the peak absorption and peak scattering wavelengths.

While the absorption and scattering peak positions in general directly correspond with another, the fwhm values do not (Figure 3D). The fwhm values are extracted from the denoised spectra by identifying the wavelength at half-maximum intensity, taking the difference between the peak position and the half-maximum, and multiplying by two to achieve a full-width. Spectra too broad to determine a half-maximum are excluded. Most NPs have a larger absorption fwhm compared to scattering, as seen by the majority markers in Figure 3D appearing below the one-to-one dashed line. This trend is consistent with the larger fwhm of the subensemble-averaged absorption spectrum in Figure 3B compared to the subensemble-averaged scattering spectrum in Figure 3A. To clarify the observed trends, optical simulations of the Mg NPs are performed and discussed next.

Optical simulations of Mg NPs elucidate why the measured absorption spectra are broader than the scattering spectra. Simulations are performed using the discrete dipole approximation (DDSCAT 7.3),^79^ and further details are included in Section S1.5 and Figure S7. We simulate the most physically reasonable NP shape using the average total length from the TEM measurements and the average shape characteristics (width and thickness) from previous characterizations of Mg NPs produced by this synthesis procedure.^28^ Specifically, we use a Wulff-inspired Mg faceted spheroid, 168 nm in length, 152 nm in width, and 128 nm in thickness with a 10 nm MgO shell (Figure 4A). We average over six light orientations to model unpolarized light. The main peak is the longitudinal, dipolar mode, with the transverse and higher order modes present beyond 500 nm. The scattering cross section (green, left axis) is approximately 1 order of magnitude greater than the absorption cross section (purple, right axis), as is expected for large NPs (Figure 4B). The absorption peak is broader than the scattering peak, reproducing the experiment. This result suggests that the difference in line width may only be due to the pure plasmonic response of the Mg NPs rather than any additional damping mechanisms. This difference in line width is significant because it indicates how lossy each observable is with respect to one another.

**Figure 4:**
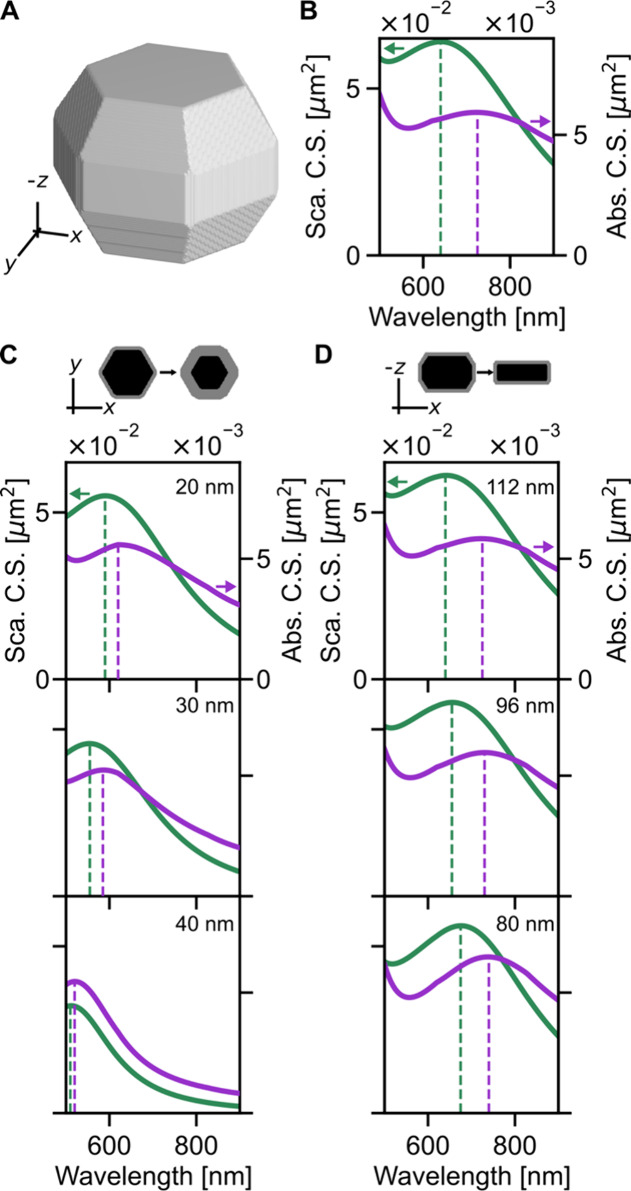
Simulations of the optical response of different Mg NP geometries offer possible explanations of experimental observations. Scattering (green, left axes) and absorption (purple, right axes) cross sections (C.S.) of a Mg NP excited with unpolarized visible light have a dipole resonance that shifts with varying NP geometry. (A−B) Simulation of a faceted spheroid with a total length (along *x*) of 168 nm, width (along *y*) of 152 nm, and a thickness of 128 nm (along *z*) and a MgO shell thickness of 10 nm. (C) Increasing the size of the MgO shell from (B) 10 nm to hypothetical thicknesses of (C) 20, 30, and 40 nm while maintaining overall NP dimensions, as indicated by the schematic with black corresponding to Mg and gray to MgO. Increasing oxidation leads to a blue shift of both peaks, with the difference between absorption and scattering maxima decreasing until they are nearly identical. Note that oxidation by trace amounts of water may cause the formation of a discontinuous Mg­(OH)_2_ (refractive index 1.6)^37^ instead of a solid MgO (refractive index 1.7) shell. For simplicity, we chose to model the latter to reveal overall trends. (D) Decreasing the Mg NP thickness in the *z* direction from (B) 128 nm to (D) 112, 96, and 80 nm while maintaining other dimensions. Decreasing NP thickness redshifts both peaks in absorption and scattering and slightly decreases their difference in peak wavelengths.

While this simulation supports the differences in line width, it does not reproduce the near-degenerate absorption and scattering peak positions as observed in experiment. We hypothesize that this mismatch is due to the measured NPs not having the precise geometry as simulated. An electron micrograph tilt series (Figure S2) indicates that the structures are faceted spheroids; however, the precise shape is variable and not an exact match to the model shown in Figure 4A. Further, there is uncertainty about the level of oxidation at the time of the experiment due to oxygen or water present in the glycerol or sample preparation. We confirm that substantial, systemic oxidation did not occur during the measurement period (Figure S5). However, small variations within the sample could lead to differences in the optical response. As such, we vary the thickness of the NP (a proxy for shape) and the oxide shell to investigate if they influence the peak position offset while maintaining a broader absorption line shape.

The first parameter we vary is the MgO layer thickness (Figure 4C). The schematic in Figure 4C illustrates the progression of shell thickness, which increases in increments of 10 nm by decreasing the diameter of the metallic core to maintain the overall NP size. As the modeled oxidation increases, both peaks blueshift but to different extents. The scattering peak shifts from 640 nm to 590 nm to 550 nm to 510 nm, while the absorption peak shifts from 725 nm to 620 nm to 580 nm to 520 nm. For a 40 nm oxide shell, the peaks are nearly degenerate, as observed in experiment. The fwhm in scattering changes from 460 nm to 380 nm to 310 nm to 230 nm while absorption changes from >650 nm to 650 nm to 400 nm to 240 nm. For the largest absorption fwhm we estimate >650 nm because higher order modes at larger energies and interband transitions in the NIR cause the peak intensity to not fully decrease to a half-maximum value. Increasing the modeled oxidation therefore leads to a reduction in fwhm in both absorption and scattering to a point where line widths become nearly identical. While increasing oxidation gives better agreement with experiment in terms of peak positions, it causes the fwhm values to become similar, opposite to experiment, while also inducing a significant blueshift that is inconsistent with the measured spectra. Thus, increased oxidation of the NPs cannot alone explain the observed experimental trends. In addition, MgO shells 20 nm and above have not been observed experimentally^25,31–35^ and are thus unlikely but are included here to demonstrate conditions when the NP peaks become degenerate.

The second parameter we vary computationally is the total thickness of the Mg NP (Figure 4D), while keeping an oxide layer thickness of 10 nm, a total length of 168 nm, and a total width of 152 nm. This size variation is implemented because the third dimension is unknown from the 2D electron microscopy images. The thickness is decreased from 128 nm in increments of 16 nm down to 80 nm, as indicated by the schematic in Figure 4D. As the thickness decreases, both peaks redshift by different degrees. The scattering peak is static at first and then increases: 640 nm to 640 nm to 655 nm to 675 nm, while the absorption peak is also static at first and then increases by a lesser extent: 725 nm to 725 nm to 730 nm to 740 nm. Decreasing the thickness therefore reduces the offset between absorption and scattering peaks while redshifting both peaks. The fwhm in scattering decreases from 460 nm to 450 nm to 420 nm to 380 nm. The fwhm in absorption appears to also decrease but could not be quantified because intensities never reached values equal or less than half-maximum. Considering that increased oxidation (Figure 4C) and decreased NP thickness (Figure 4D) both reduce the offset between peak maxima and maintain broader absorption line widths, consistent with experiment and that the former blueshifts the spectrum while the latter redshifts the spectrum, the actual experimental NP morphology could be a combination of the two explored conditions, i.e., thinner and slightly more oxidized because of glycerol needed for PTA measurements.

To further understand why absorption is broader than scattering and why there is a peak offset between absorption and scattering for these Mg NPs, we incrementally reduce the simulation complexity to isolate the physical origin of the broadening and offset using Mie theory for Mg nanospheres of similar size. These simulations are not meant to represent the specific faceted NPs measured but instead serve to elucidate the trends in line shape. We first test whether the presence of the MgO shell alone could cause both phenomena, using an extreme approach by varying the refractive index of the infinite medium surrounding the NPs from *n* = 1 (air) to *n* = 1.7 (MgO). In air, the peaks are already offset and absorption is broader. Increasing the refractive index of the background redshifts both peaks, widens the offset, and further broadens absorption (Figure S8). Thus, the oxide shell likely contributes to but cannot fully explain the observed trends of the peak offset and broader absorption. To understand why even just pure Mg nanospheres in air support the peak offset and broader absorption, we simplified the model from considering Mie modes ( l ) up to l=10 (Figure S9A–D) to only l=1 (Figure S9E–H). Again, the dipolar response of Mg nanospheres already yields both trends, thus confirming that they are not caused by higher order modes. We next fit a Drude model to the Mg dielectric data and find that by isolating the Drude response, excluding Mg’s near-infrared interband transitions, both trends persist (Figure S9I,K). When we do the same analysis using Au dielectric data, we find that the absorption and scattering peaks are nearly degenerate with minimal absorption broadening (Figure S9J,L). For smaller 100 nm nanospheres, there is no offset in peaks for Mg (or Au), although the larger absorption line width remains. We therefore conclude that the peak offset occurs as the NP size increases irrespective of higher order modes and that the broadening is a result of the Drude part of the dielectric function modifying the lowest order dipole mode. Finally, by varying the Drude model parameters, we identify the plasma frequency and consequently the larger real part of the dielectric function as the origin of both peak offset and absorption broadening for Mg. This behavior is a unique optical feature of Mg and is likely present for other metals with large, dispersive real dielectric functions (e.g., Al) in contrast to metals with small real dielectric functions (e.g., Au, Ag).^8^ Figure S10 summarizes the emergence of these trends. Related studies on other metals (Au, Ag, Al) have confirmed that it is the dispersion of the real part of the dielectric function that influences plasmon damping.^80,81^

In conclusion, we characterized the absorption and scattering by Mg faceted spheroids at the single-particle level and determined that, despite their broad ensemble extinction spectrum, these NPs support size- and shape-tunable resonances in absorption and scattering caused by localized surface plasmons. Across most measured Mg NPs, the absorption and scattering peaks occur at the same wavelength, but absorption is significantly broader than scattering. Optical simulations reproduce these trends and indicate that the broader absorption is due to the size of the NP, the presence of a MgO shell, and the large real dielectric function of Mg. The differences in line shape between absorption and scattering underscore the importance of measuring both quantities rather than relying on scattering as an indicator of the absorption peak position and line width. As we demonstrated here, inferring an absorption spectrum from a measured scattering spectrum likely leads to inaccuracies. In fact, complete optical characterization of plasmonic NPs requires a combination of single-particle absorption and scattering spectroscopy together with correlated electron imaging as well as electromagnetic simulations. Our results have important implications when proper characterization of the light absorption by plasmonic NPs is required, as is the case for many applications of plasmonic nanoparticles ranging from photothermal therapy to plasmon-assisted catalysis, which all depend on absorption and not scattering.^49,50,52–54^

## Supplementary Material



## Notes

Mg NP synthesis, sample preparation for single-particle measurements, DFS and PTA spectroscopy measurements, simulation details, additional TEM images, ensemble extinction measurements, DFS measurements before and after PTA, all correlated PTA/DFS/SEM data, Mie theory modeling, and dielectric analysis (PDF)

## References

[1] Ezendam S., Herran M., Nan L., Gruber C., Kang Y., Gröbmeyer F., Lin R., Gargiulo J., Sousa-Castillo A., Cortés E. (2022). Hybrid Plasmonic Nanomaterials for Hydrogen Generation
and Carbon Dioxide Reduction. ACS Energy Lett..

[2] Sayed M., Yu J., Liu G., Jaroniec M. (2022). Non-Noble Plasmonic Metal-Based Photocatalysts. Chem. Rev..

[3] Lin L. L., Alvarez-Puebla R., Liz-Marzán L.
M., Trau M., Wang J., Fabris L., Wang X., Liu G., Xu S., Han X. X., Yang L., Shen A., Yang S., Xu Y., Li C., Huang J., Liu S.-C., Huang J.-A., Srivastava I., Li M., Tian L., Nguyen L. B. T., Bi X., Cialla-May D., Matousek P., Stone N., Carney R. P., Ji W., Song W., Chen Z., Phang I. Y., Henriksen-Lacey M., Chen H., Wu Z., Guo H., Ma H., Ustinov G., Luo S., Mosca S., Gardner B., Long Y.-T., Popp J., Ren B., Nie S., Zhao B., Ling X. Y., Ye J. (2025). Surface-Enhanced Raman
Spectroscopy for Biomedical Applications: Recent Advances and Future
Challenges. ACS Appl. Mater. Interfaces.

[4] Le
Ru E. C., Grand J., Sow I., Somerville W. R. C., Etchegoin P. G., Treguer-Delapierre M., Charron G., Félidj N., Lévi G., Aubard J. (2011). A Scheme for Detecting Every Single
Target Molecule with Surface-Enhanced Raman Spectroscopy. Nano Lett..

[5] Cao J., Sun T., Grattan K. T. V. (2014). Gold
Nanorod-Based Localized Surface
Plasmon Resonance Biosensors: A Review. Sens.
Actuators B Chem..

[6] Hu J.-J., Cheng Y.-J., Zhang X.-Z. (2018). Recent Advances
in Nanomaterials
for Enhanced Photothermal Therapy of Tumors. Nanoscale.

[7] Lalisse A., Tessier G., Plain J., Baffou G. (2015). Quantifying the Efficiency
of Plasmonic Materials for Near-Field Enhancement and Photothermal
Conversion. J. Phys. Chem. C.

[8] Hopper E. R., Boukouvala C., Asselin J., Biggins J. S., Ringe E. (2022). Opportunities
and Challenges for Alternative Nanoplasmonic Metals: Magnesium and
Beyond. J. Phys. Chem. C.

[9] Kim S., Kim J., Park J., Nam J. (2018). Nonnoble-Metal-Based Plasmonic Nanomaterials:
Recent Advances and Future Perspectives. Adv.
Mater..

[10] Jung I., Lee S., Lee S., Kim J., Kwon S., Kim H., Park S. (2025). Colloidal Synthesis of Plasmonic Complex Metal Nanoparticles: Sequential
Execution of Multiple Chemical Toolkits Increases Morphological Complexity. Chem. Rev..

[11] Scarabelli L., Sun M., Zhuo X., Yoo S., Millstone J. E., Jones M. R., Liz-Marzán L.
M. (2023). Plate-Like Colloidal
Metal Nanoparticles. Chem. Rev..

[12] Kasani S., Curtin K., Wu N. (2019). A Review of 2D and 3D Plasmonic Nanostructure
Array Patterns: Fabrication, Light Management and Sensing Applications. Nanophotonics.

[13] Hsu S.-W., Rodarte A. L., Som M., Arya G., Tao A. R. (2018). Colloidal
Plasmonic Nanocomposites: From Fabrication to Optical Function. Chem. Rev..

[14] Pastoriza-Santos I., Kinnear C., Pérez-Juste J., Mulvaney P., Liz-Marzán L.
M. (2018). Plasmonic
Polymer Nanocomposites. Nat. Rev. Mater..

[15] Coviello, V. ; Forrer, D. ; Amendola, V. Recent Developments in Plasmonic Alloy Nanoparticles: Synthesis, Modelling, Properties and Applications. ChemPhysChem 2022, 23 (21). 10.1002/cphc.202200136. PMC9804694 35502819

[16] Kar N., Skrabalak S. E. (2025). Synthetic Methods for High-Entropy Nanomaterials. Nat. Rev. Mater..

[17] Ha M., Kim J.-H., You M., Li Q., Fan C., Nam J.-M. (2019). Multicomponent Plasmonic Nanoparticles:
From Heterostructured
Nanoparticles to Colloidal Composite Nanostructures. Chem. Rev..

[18] King M. E., Fonseca Guzman M. V., Ross M. B. (2022). Material Strategies for Function
Enhancement in Plasmonic Architectures. Nanoscale.

[19] Sterl F., Linnenbank H., Steinle T., Mörz F., Strohfeldt N., Giessen H. (2018). Nanoscale Hydrogenography on Single
Magnesium Nanoparticles. Nano Lett..

[20] Shin C.-H., Lee H.-Y., Gyan-Barimah C., Yu J.-H., Yu J.-S. (2023). Magnesium:
Properties and Rich Chemistry for New Material Synthesis and Energy
Applications. Chem. Soc. Rev..

[21] Duan X., Liu N. (2019). Magnesium for Dynamic
Nanoplasmonics. Acc.
Chem. Res..

[22] Jeong H.-H., Mark A. G., Fischer P. (2016). Magnesium Plasmonics
for UV Applications
and Chiral Sensing. Chem. Commun..

[23] Jahnen-Dechent W., Ketteler M. (2012). Magnesium
Basics. Clin. Kidney
J..

[24] Sterl F., Strohfeldt N., Walter R., Griessen R., Tittl A., Giessen H. (2015). Magnesium
as Novel Material for Active Plasmonics in
the Visible Wavelength Range. Nano Lett..

[25] Hopper E. R., Wayman T. M. R., Asselin J., Pinho B., Boukouvala C., Torrente-Murciano L., Ringe E. (2022). Size Control in the Colloidal Synthesis
of Plasmonic Magnesium Nanoparticles. J. Phys.
Chem. C.

[26] Ten A., Boukouvala C., Lomonosov V., Ringe E. (2025). Colloidal Synthesis
and Etching Yield Monodisperse Plasmonic Quasi-Spherical Mg Nanoparticles. Nanoscale Horiz..

[27] Wayman T.
M. R., Lomonosov V., Ringe E. (2024). Capping Agents Enable Well-Dispersed
and Colloidally Stable Metallic Magnesium Nanoparticles. J. Phys. Chem. C.

[28] Lomonosov V., Hopper E. R., Ringe E. (2023). Seed-Mediated
Synthesis of Monodisperse
Plasmonic Magnesium Nanoparticles. Chem. Commun..

[29] Abbas D., Naciri A. E., Bouzourâa M., Alhaddad T., Kassem A., Bouché A., Akil S. (2025). Unleashing the Potential of Magnesium
Nanoparticles: A Green Synthesis for Sustainable Sensing Solutions. Sustain. Mater. Technol..

[30] Ritschel C., Donsbach C., Feldmann C. (2024). Reactive Magnesium
Nanoparticles
to Perform Reactions in Suspension. Chem. -
Eur. J..

[31] Ringe E. (2020). Shapes, Plasmonic
Properties, and Reactivity of Magnesium Nanoparticles. J. Phys. Chem. C.

[32] Lomonosov V., Yang J., Fan Y., Hofmann S., Ringe E. (2024). Stability
of Plasmonic Mg-MgO Core-Shell Nanoparticles in Gas-Phase Oxidative
Environments. Nano Lett..

[33] Biggins J. S., Yazdi S., Ringe E. (2018). Magnesium
Nanoparticle Plasmonics. Nano Lett..

[34] Boukouvala C., West C. A., Ten A., Hopper E., Ramasse Q. M., Biggins J. S., Ringe E. (2024). Far-Field, near-Field and Photothermal
Response of Plasmonic Twinned Magnesium Nanostructures. Nanoscale.

[35] Ten A., Lomonosov V., Boukouvala C., Ringe E. (2024). Magnesium Nanoparticles
for Surface-Enhanced Raman Scattering and Plasmon-Driven Catalysis. ACS Nano.

[36] Asselin J., Boukouvala C., Hopper E. R., Ramasse Q. M., Biggins J. S., Ringe E. (2020). Tents, Chairs,
Tacos, Kites, and Rods: Shapes and Plasmonic Properties
of Singly Twinned Magnesium Nanoparticles. ACS
Nano.

[37] Asselin J., Hopper E. R., Ringe E. (2021). Improving the Stability of Plasmonic
Magnesium Nanoparticles in Aqueous Media. Nanoscale.

[38] Asselin, J. ; Boukouvala, C. ; Wu, Y. ; Hopper, E. R. ; Collins, S. M. ; Biggins, J. S. ; Ringe, E. Decoration of Plasmonic Mg Nanoparticles by Partial Galvanic Replacement. J. Chem. Phys. 2019, 151 (24). 10.1063/1.5131703. 31893891

[39] Lomonosov V., Wayman T. M. R., Hopper E. R., Ivanov Y. P., Divitini G., Ringe E. (2023). Plasmonic Magnesium Nanoparticles Decorated with Palladium Catalyze
Thermal and Light-Driven Hydrogenation of Acetylene. Nanoscale.

[40] Patil S. J., Lomonosov V., Ringe E., Kurouski D. (2023). Tip-Enhanced Raman
Imaging of Plasmon-Driven Coupling of 4-Nitrobenzenethiol on Au-Decorated
Magnesium Nanostructures. J. Phys. Chem. C.

[41] West C. A., Lomonosov V., Pehlivan Z. S., Ringe E. (2023). Plasmonic Magnesium
Nanoparticles Are Efficient Nanoheaters. Nano
Lett..

[42] Knobeloch M., O’Dell Z. J., Edwards M. E., Huang C., Nguyen M., Wahab O. J., Baker L. A., Henkelman G., Ye X., Yan X., Willets K. A., Skrabalak S. E. (2025). Learning
from Metal Nanocrystal Heterogeneity: A Need for Information-Rich
and High-Throughput Single-Nanocrystal Measurements. ACS Nanosci. Au.

[43] O’Dell Z. J., Knobeloch M., Skrabalak S. E., Willets K. A. (2024). High-Throughput
All-Optical Determination of Nanorod Size and Orientation. Nano Lett..

[44] Sridhar S., Nikolov M. E., Beutler E. K., Knobeloch M., Paranzino B., Vernon K. L., Zhong Y., Ye X., Baker L. A., Skrabalak S. E., Masiello D. J., Willets K. A. (2025). Scattering
vs Interference in Interferometric Scattering Spectroscopy of Plasmonic
Nanoparticles. J. Phys. Chem. Lett..

[45] Rodríguez-Fernández J., Novo C., Myroshnychenko V., Funston A. M., Sánchez-Iglesias A., Pastoriza-Santos I., Pérez-Juste J., García De Abajo F. J., Liz-Marzán L. M., Mulvaney P. (2009). Spectroscopy, Imaging, and Modeling
of Individual Gold Decahedra. J. Phys. Chem.
C.

[46] Collins S. S. E., Cittadini M., Pecharromán C., Martucci A., Mulvaney P. (2015). Hydrogen Spillover
between Single Gold Nanorods and Metal Oxide Supports: A Surface Plasmon
Spectroscopy Study. ACS Nano.

[47] Nguyen T. N., Cappillino P. J., Chang W.-S. (2025). Revealing Enhanced Size Uniformity
of the Electrochemical Deposition of Palladium Nanoparticles via Single-Particle
Dark-Field Scattering Imaging. J. Phys. Chem.
C.

[48] Menghrajani K. S., McLaren F., Maier S. A., Sastry M. (2025). Structure-Property
Analysis of Bimetallic Plasmonic Nanoparticles Using Correlative Optical
and Electron Microscopy. J. Phys. Chem. C.

[49] Brooks J. L., Warkentin C. L., Saha D., Keller E. L., Frontiera R. R. (2018). Toward
a Mechanistic Understanding of Plasmon-Mediated Photocatalysis. Nanophotonics.

[50] Hu Y., Wijerathne N., Pabel M. Y., Arachchige D. K., Wei W. D. (2025). Manipulating Plasmon-Generated
Hot Carriers for Photocatalysis. Acc. Chem.
Res..

[51] Herran M., Sousa-Castillo A., Fan C., Lee S., Xie W., Döblinger M., Auguié B., Cortés E. (2022). Tailoring
Plasmonic Bimetallic Nanocatalysts Toward Sunlight-Driven H_2_ Production. Adv. Funct. Mater..

[52] Baffou G., Cichos F., Quidant R. (2020). Applications
and Challenges of Thermoplasmonics. Nat. Mater..

[53] Ruhoff V. T., Arastoo M. R., Moreno-Pescador G., Bendix P. M. (2024). Biological Applications
of Thermoplasmonics. Nano Lett..

[54] Yang, B. ; Li, C. ; Wang, Z. ; Dai, Q. Thermoplasmonics in Solar Energy Conversion: Materials, Nanostructured Designs, and Applications. Adv. Mater. 2022, 34 (26). 10.1002/adma.202107351. 35271744

[55] Wang Q., Ren Z.-H., Zhao W.-M., Wang L., Yan X., Zhu A., Qiu F., Zhang K.-K. (2022). Research Advances on Surface Plasmon
Resonance Biosensors. Nanoscale.

[56] Jeon H. B., Tsalu P. V., Ha J. W. (2019). Shape Effect
on the Refractive Index
Sensitivity at Localized Surface Plasmon Resonance Inflection Points
of Single Gold Nanocubes with Vertices. Sci.
Rep..

[57] Schlücker S. (2014). Surface-Enhanced
Raman Spectroscopy: Concepts and Chemical Applications. Angew. Chem., Int. Ed..

[58] Jain P. K., Lee K. S., El-Sayed I. H., El-Sayed M. A. (2006). Calculated Absorption
and Scattering Properties of Gold Nanoparticles of Different Size,
Shape, and Composition: Applications in Biological Imaging and Biomedicine. J. Phys. Chem. B.

[59] Van
Dijk M. A., Tchebotareva A. L., Orrit M., Lippitz M., Berciaud S., Lasne D., Cognet L., Lounis B. (2006). Absorption
and Scattering Microscopy of Single Metal Nanoparticles. Phys. Chem. Chem. Phys..

[60] Fonseca
Guzman M. V., Ross M. B. (2021). Radiative Contributions Dominate
Plasmon Broadening for Post-Transition Metals in the Ultraviolet. J. Phys. Chem. C.

[61] De
Aberasturi D. J., Serrano-Montes A. B., Liz-Marzán L. M. (2015). Modern
Applications of Plasmonic Nanoparticles: From Energy to Health. Adv. Opt. Mater..

[62] Evanoff D. D., Chumanov G. (2004). Size-Controlled Synthesis
of Nanoparticles. 2. Measurement
of Extinction, Scattering, and Absorption Cross Sections. J. Phys. Chem. B.

[63] Bilankohi S. (2015). Optical Scattering
and Absorption Characteristics of Silver and Silica/Silver Core/Shell
Nanoparticles. Orient. J. Chem..

[64] Etchegoin P. G., Le Ru E. C., Meyer M. (2006). An Analytic Model for the Optical
Properties of Gold. J. Chem. Phys..

[65] Sundararaman R., Narang P., Jermyn A. S., Goddard W. A., Atwater H. A. (2014). Theoretical Predictions
for Hot-Carrier Generation
from Surface Plasmon Decay. Nat. Commun..

[66] Yuan T., Guo X., Lee S. A., Brasel S., Chakraborty A., Masiello D. J., Link S. (2025). Chemical Interface
Damping Revealed
by Single-Particle Absorption Spectroscopy. ACS Nano.

[67] Yorulmaz M., Nizzero S., Hoggard A., Wang L. Y., Cai Y. Y., Su M. N., Chang W. S., Link S. (2015). Single-Particle Absorption
Spectroscopy by Photothermal Contrast. Nano
Lett..

[68] He G. S., Zhu J., Yong K.-T., Baev A., Cai H.-X., Hu R., Cui Y., Zhang X.-H., Prasad P. N. (2010). Scattering and Absorption Cross-Section
Spectral Measurements of Gold Nanorods in Water. J. Phys. Chem. C.

[69] Le Ru, E. ; Etchegoin, P. Principles of Surface-Enhanced Raman Spectroscopy and Related Plasmonic Effects, 1st ed.; Elsevier Science: San Diego, CA, 2008.

[70] Zhang Y. J. (2011). Investigation
of Gold and Silver Nanoparticles on Absorption Heating and Scattering
Imaging. Plasmonics.

[71] Miller M. M., Lazarides A. A. (2006). Sensitivity
of Metal Nanoparticle Plasmon Resonance
Band Position to the Dielectric Environment as Observed in Scattering. J. Opt. Pure Appl. Opt..

[72] Danan Y., Ramon Y., Azougi J., Douplik A., Zalevsky Z. (2015). Decoupling
and Tuning the Light Absorption and Scattering Resonances in Metallic
Composite Nanostructures. Opt. Express.

[73] Zhu J., Li J., Zhao J. (2011). Tuning the
Wavelength Drift between Resonance Light
Absorption and Scattering of Plasmonic Nanoparticle. Appl. Phys. Lett..

[74] Adhikari S., Spaeth P., Kar A., Baaske M. D., Khatua S., Orrit M. (2020). Photothermal Microscopy: Imaging the Optical Absorption of Single
Nanoparticles and Single Molecules. ACS Nano.

[75] Berciaud, S. ; Cognet, L. ; Blab, G. A. ; Lounis, B. Photothermal Heterodyne Imaging of Individual Nonfluorescent Nanoclusters and Nanocrystals. Phys. Rev. Lett. 2004, 93 (25). 10.1103/PhysRevLett.93.257402. 15697940

[76] Gaiduk A., Ruijgrok P. V., Yorulmaz M., Orrit M. (2010). Detection Limits in
Photothermal Microscopy. Chem. Sci..

[77] Ringe E., Langille M. R., Sohn K., Zhang J., Huang J., Mirkin C. A., Van Duyne R. P., Marks L. D. (2012). Plasmon Length:
A Universal Parameter to Describe Size Effects in Gold Nanoparticles. J. Phys. Chem. Lett..

[78] Noguez C. (2007). Surface Plasmons
on Metal Nanoparticles: The Influence of Shape and Physical Environment. J. Phys. Chem. C.

[79] Draine B. T., Flatau P. J. (1994). Discrete-Dipole
Approximation for Scattering Calculations. J.
Opt Soc. Am. A.

[80] Ross M. B., Schatz G. C. (2015). Radiative Effects
in Plasmonic Aluminum and Silver
Nanospheres and Nanorods. J. Phys. Appl. Phys..

[81] Schmucker A. L., Harris N., Banholzer M. J., Blaber M. G., Osberg K. D., Schatz G. C., Mirkin C. A. (2010). Correlating
Nanorod Structure with
Experimentally Measured and Theoretically Predicted Surface Plasmon
Resonance. ACS Nano.

