# Supplementary material for: Resolving Single-Particle Absorption and Scattering by Plasmonic Magnesium Nanoparticles
Source: Nano Lett. 2026 Apr 1;26(14):4774–81. doi: 10.1021/acs.nanolett.6c00484 (PMC13088366; doi:10.1021/acs.nanolett.6c00484)
Supplement: Supplementary file 1 [file nl6c00484_si_001.pdf]

SUPPORTING INFORMATION

Resolving Single-Particle Absorption and Scattering by Plasmonic Magnesium Nanoparticles

Claire A. West[1], Tinglian Yuan[1], Tathagata Chatterjee[1], Vladimir Lomonosov[2], Jae-Ho Kim[1], Emilie Ringe[2]*, Stephan Link[1]*

1. Department of Chemistry, Department of Electrical and Computer Engineering, Materials Research Lab, University of Illinois Urbana-Champaign, Urbana, Illinois 61801, United States
2. Department of Earth Sciences, University of Cambridge, Downing Street, Cambridge CB2 3EQ, United Kingdom and Department of Materials Science and Metallurgy, University of Cambridge, 27 Charles Babbage Road, Cambridge CB3 0FS, United Kingdom

S1. Methods

S1.1 Mg NP synthesis

Li pellets (99%), naphthalene, biphenyl, 1.0 M di-n-butylmagnesium in heptane, 0.7M n-butyl-sec-butylmagnesium poly(vinyl pyrrolidone) (PVP, MW 10,000), anhydrous tetrahydrofuran (THF) and anhydrous isopropanol (IPA) were purchased from Sigma-Aldrich and used as supplied. All glassware was washed with aqua regia and flame-dried under vacuum.

Mg NPs were synthesized using a seed-mediated growth approach.[1] L_2Napht or Li_2BiPhenyl dianions were used as reducing agents. For Li_2Napht: 0.028 g Li pellets (4.05 mmol), 0.26 g naphthalene (2.03 mmol), 20 mg PVP (0.18 mmol monomer), and 10.75 mL degassed anhydrous THF were added to a 25 mL Schlenk flask under Ar atmosphere and sonicated for 45 minutes. For Li_2BiPhenyl: the same protocol was followed using 0.32g biphenyl instead of naphthalene. The "seeds" were synthesized using a one-step reduction of di-n-butylmagnesium with Li_2Biphenyl. Mg NP "seed" synthesis was initiated by injecting 1.75 mL di-n-$MgBu_2$ in heptane (1.0 M) into freshly prepared Li_2Napht solution followed by addition of 2 mL naphthalene in THF (1.0 M) after 5 minutes of reaction, to convert all unreacted Li_2Napht to LiNapht. The resulting mixture reacted for 60 minutes, quenched with 2 mL IPA. The size of the spheroids was increased by addition of a second identical dose of Mg precursor 30 minutes after the naphthalene injection and letting the mixture react for a further 18 hours. In all syntheses the solid product was recovered by centrifugation, and residual by-products were removed by centrifugation and redispersion steps in anhydrous IPA twice, anhydrous THF twice, and anhydrous IPA twice, before redispersing in anhydrous IPA.

S1.2 Sample preparation for single-particle measurements

Mg NPs were deposited onto a Au-patterned coverslip by spin-coating. Coverslips were patterned to correlate individual NPs between measurements, and were made by evaporating Au over a windowed TEM grid taped on the glass coverslip. The sample was assembled into a

sandwich geometry (Fig. 2A) by adding 90 μL of degassed glycerol, a silicone spacer (0.5 mm thick), and a top coverslip. SEM was performed last to not damage the NPs. The sandwich sample was disassembled and washed with IPA to remove glycerol and dried with N_2 gas. An approximate 6 nm layer of carbon was evaporated onto the washed sample to create a conductive layer to enable SEM imaging.

S1.3 DFS spectroscopy

Hyperspectral dark-field scattering measurements were taken on an inverted microscope (Zeiss, Axio Observer.D1m) with a halogen lamp (Zeiss, HAL 100) focused by an oil-immersion condenser in the dark-field geometry ("D" setting, numerical aperture NA=1.4). The scattered light was collected with a reflective objective (Beck Optronics Solutions, 74x, NA = 0.65). Although a reflective objective was used to minimize chromatic aberrations, we cannot completely rule out residual chromatic aberrations and therefore exclude that the true scattering spectra could be slightly broader than reported here. Considering that our simulations reproduced the experimental trends well, we expect a systematic underestimation of the scattering linewidth to be overall small though. The light then passed through a spectrograph (Princeton Instruments, Acton SP2150i), composed of a 20 μm slit, a diffraction grating (300 lines/mm) centered at 650 nm, and a charge-coupled (CCD) camera (Princeton Instruments, PIXIS 400BR). All components were mounted on a motorized translation stage. Exposure time was 3 seconds. Background subtraction and white light lamp correction was performed in the conventional manner:[2] $I = (I_{\mathrm{meas}} - I_{\mathrm{back}})/(I_{\mathrm{lamp}} - I_{\mathrm{dark}})$ where I_{meas} was the average of the 3x3 grid (9 pixels) containing the NP signal, I_{back} was the local background calculated by finding the minimum of four averaged 3x3 grids surrounding but outside the NP, I_{lamp} was the intensity from the halogen lamp, and I_{dark} was the intensity with no lamp light. To extract peak position and FWHM of the spectra, Gaussian blur was used with the standard deviation of the Gaussian filter set to 10.

S1.4 PTA spectroscopy

Single-particle absorption measurements were performed in a transmission-based photothermal imaging and spectroscopy setup.[3] A commercial inverted microscope (Zeiss, Axio Observer.D1) housed two objectives: a high NA objective (Zeiss, Plan-Apochromat 63×/NA=1.4) to focus the lasers onto the sample and a second objective (Zeiss, LP NeoFluar 40×/NA=0.6) to collect the transmitted light. The pump laser (NKT Photonics, SuperK FIU-15) was fiber-coupled into an acousto-optic tunable filter with two channels (Fianium, SELECT VIS and NIR1 AOTFs). The spectral ranges of these channels were 550-750 nm (visible) and 650-1000 nm (NIR), respectively, and were stitched together in post-processing. We employed a step size of 10 nm. The laser was modulated by a function generator (FLUKE, 271 10 MHz DDS) providing a 30 kHz square wave. The pump was focused at the sample plane with a power of approximately 24 μW for the visible channel (at 700 nm), and 21 μW for the NIR channel (at 700 nm). The 532 nm probe laser (Coherent, OBIS532 LS 50 mW) was focused 0.9 μm above the sample plane, with a power of 225 μW (1×10^9 W/m^2). A silicon-based photodetector (FEMTO, HCA-S-200M-Si) received the transmitted probe light. The electrical voltage converted from the received photons was sent out to a pre-amplifier (FEMTO, DHPCA-100, AC 20 dB) and then a lock-in amplifier (Stanford Research Systems, SR844) for signal demodulation.

Each NP absorption spectrum was the average of two experiments with the linearly polarized heating beam polarized in two orthogonal directions. Chromatic aberrations and variable pump laser powers were corrected for in post-processing, using the previously developed calibration method that relies on recording the absorption spectrum from the thin gold film present as marker grid for particle correlation with SEM.[3] Prior to spectroscopy measurements, we performed photothermal imaging with the heating laser wavelength at 700 nm for each region of interest to locate particle positions.

Lastly, we established the following protocols to determine whether to discard NP measurements due to any changes to the NP over the course of the measurements. PTA on each

NP was measured multiple times. If the spectrum continuously changed from measurement to measurement, it was discarded. There were some NPs that showed an initial change in PTA lineshape but then stabilized and did not change upon repeat measurements. This data was included in the overall analysis. DFS was performed before and after PTA (see Fig. S4). Post-PTA DFS spectra were used for the analysis in the main text to ensure that if initial NP degradation occurred and then stabilized, it would not be reflected in the difference between PTA and DFS spectra. To extract the peak position and FWHM, Gaussian blur was used with the standard deviation of the Gaussian filter set to 1.

S1.5 Simulation details

Optical simulations were performed using the discrete dipole approximation (DDSCAT 7.3)[4]. The NP shapes were modeled using a modified Wulff construction (Crystal Creator[5]). The dielectric data for Mg was from Palik.[6] A refractive index of 1.7 was used for the MgO shell, and the surrounding medium was modelled as infinite with an effective refractive index of 1.5, obtained by averaging the glass substrate and glycerol. We did not expect that explicitly including a substrate in the calculations would impact observed trends. The reported spectra were an average of six light orientations to model unpolarized light. Parameters for all simulations used to create the NP shape in Crystal Creator were single crystal hexagonal and 1 nm dipole spacing. The energy / growth velocities used for the original shape (Fig. 4A) were 0001: 33.5, $10\bar{1}0$: 41, $10\bar{1}1$: 41, $11\bar{2}0$: 46. To extract peak positions, we determined the wavelength corresponding to the maximum of the lowest order mode. To extract FWHM, we calculated the wavelength to the red of the peak that corresponds to half the value at the peak and multiplied by two (full-width). For some simulations, the peak did not decay to half maximum because of interband transitions.

S2. Additional measurements and calculations

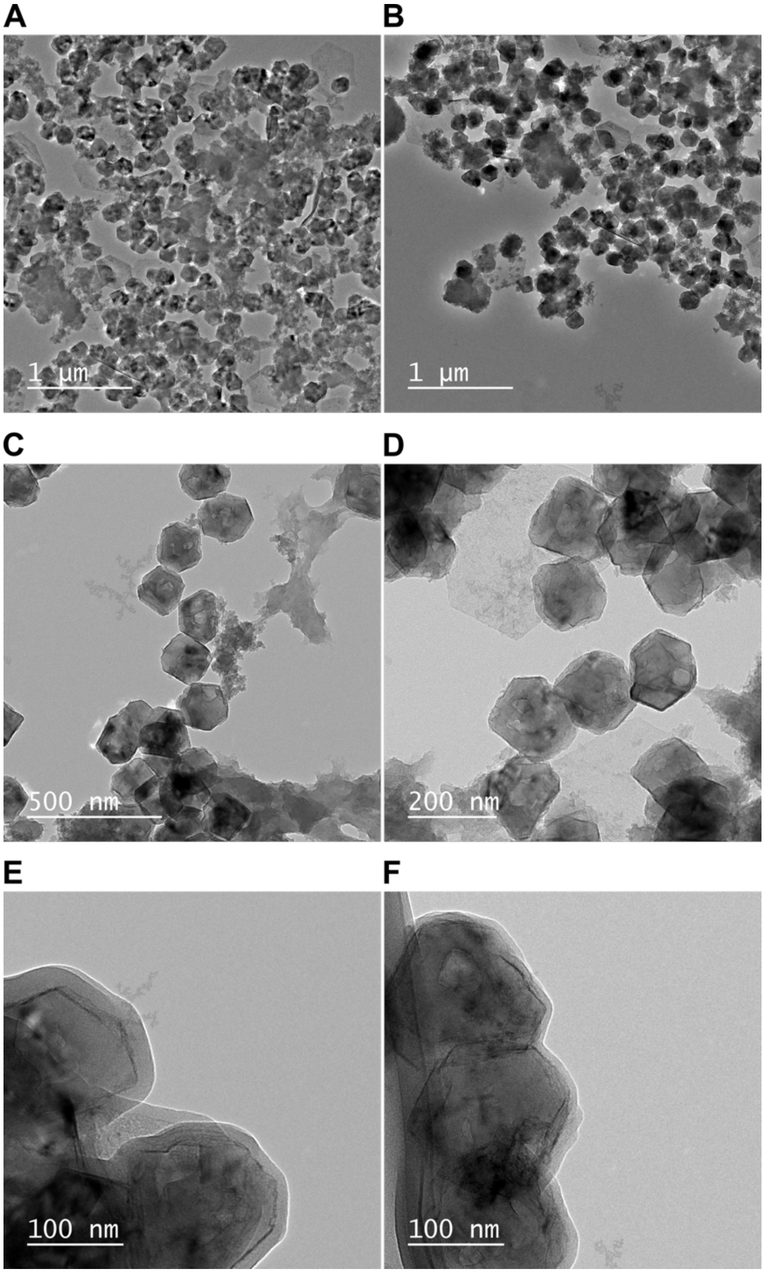

Figure S1. Additional TEM images of Mg NPs. (A-B) Large and (C-D) medium scale fields of view of Mg NPs. (E-F) The oxide and organic layer surrounding Mg NPs is visible.

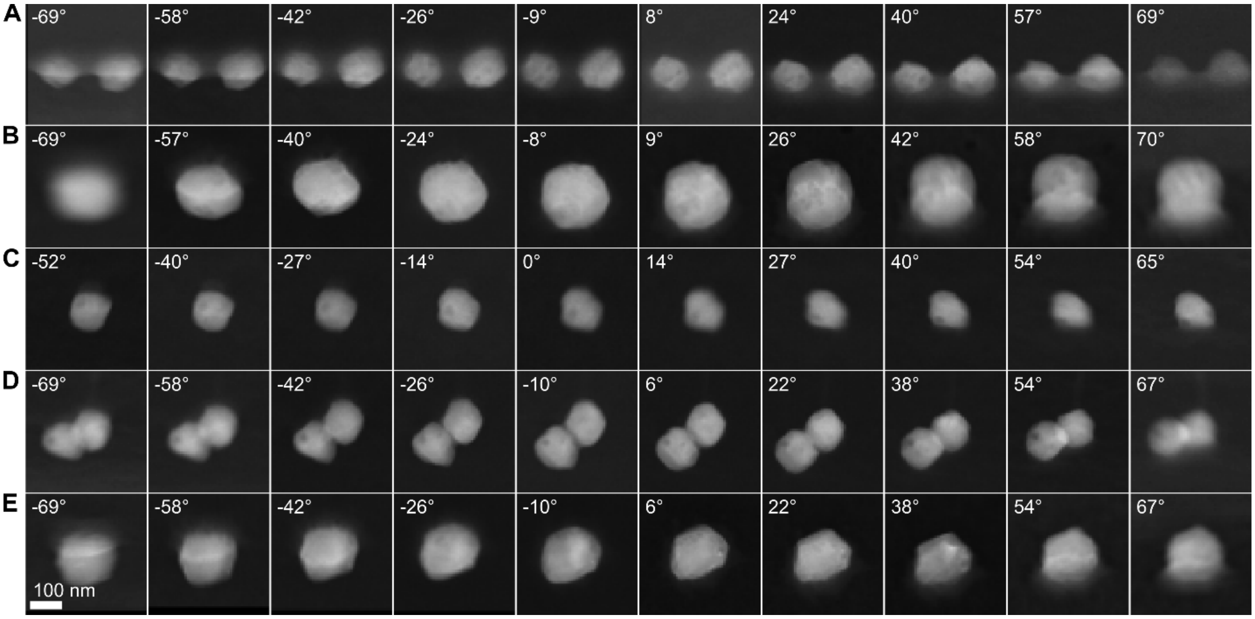

Figure S2. (A-E) High-angle annular dark-field scanning transmission electron microscopy (HAADF-STEM) tilt series of representative Mg NPs acquired over tilt ranges from approximately -70° to +70°. Each row is a different Mg NP, and the scale bar is consistent across all images. Tilt series confirm the facetted spheroid morphology of the Mg NPs.

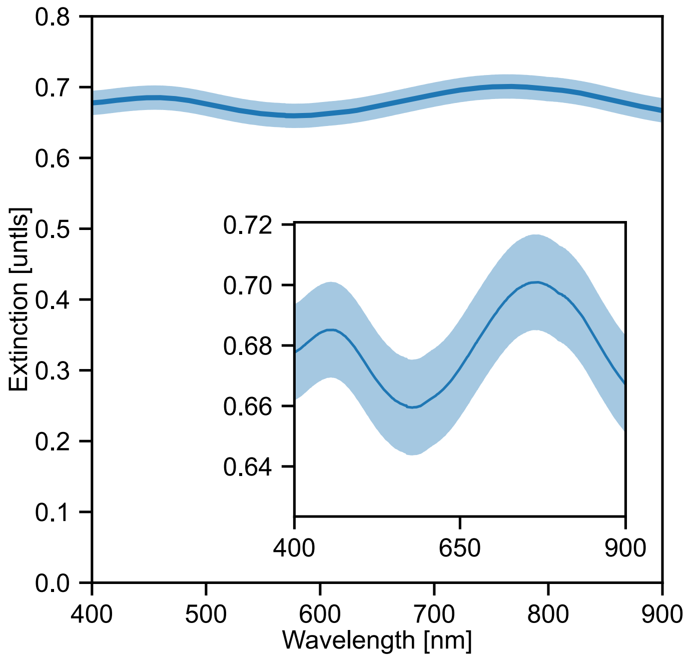

Figure S3. Ensemble extinction spectroscopy of the colloidal Mg NP solution. The solid blue line is reproduced from main text Figure 1C and is the average of 120 independent measurements of the colloid. Each individual spectrum is normalized by setting the maximum value of the spectrum to one, and then averaged. Shaded blue is the standard deviation among the 120 normalized spectra. The inset is a zoom in along the y-axis, and reveals two faint bumps in the spectra.

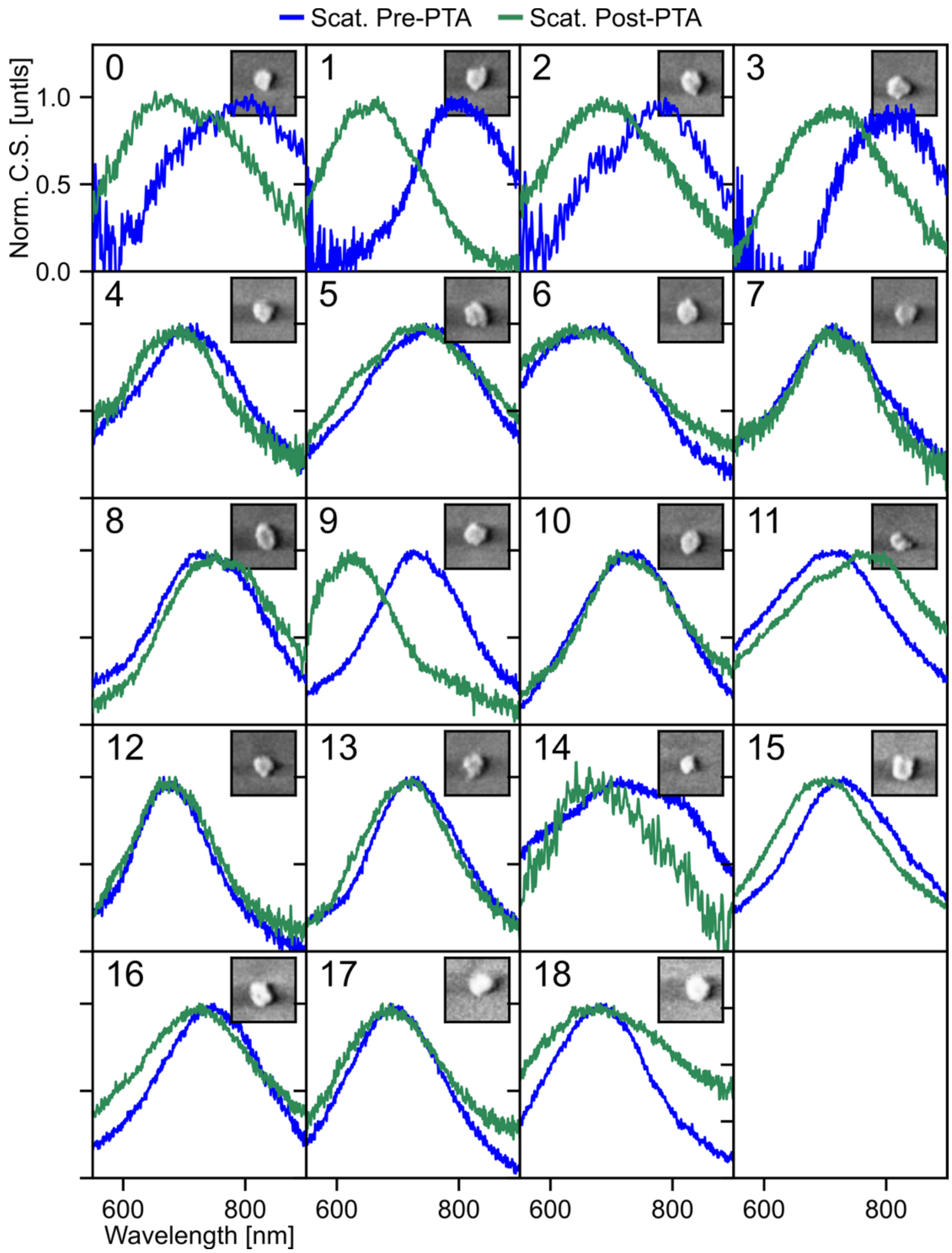

Figure S4. DFS measurements before and after PTA spectroscopy. Blue traces are scattering spectra measured before PTA spectroscopy and green traces are scattering spectra measured afterwards. All spectra are normalized. SEM images (measured after post-PTA scattering) are inset in each panel with a panel width of 490 nm. Most NPs underwent no spectral changes caused by PTA (*i.e.*, NPs 4-8, 10-13, 15-17), and the absence of peak shifts and broadening for those NPs provides evidence that Mg NPs are stable under laser irradiation. Among the

NPs with a change, the difference in spectra was a blue-shift and / or slight spectral broadening. This change could be due to the NPs oxidizing caused by laser illumination during PTA spectroscopy or NP reshaping. The NPs that underwent the most prominent changes (NPs 0-3) were from the same region of the sample. We did not discard the NPs that changed after PTA spectroscopy because they stabilized during the PTA measurement, and post-DFS was done immediately (~24 hours) afterwards. Scattering spectra reported in main text are the ones acquired post-PTA to ensure most appropriate comparisons.

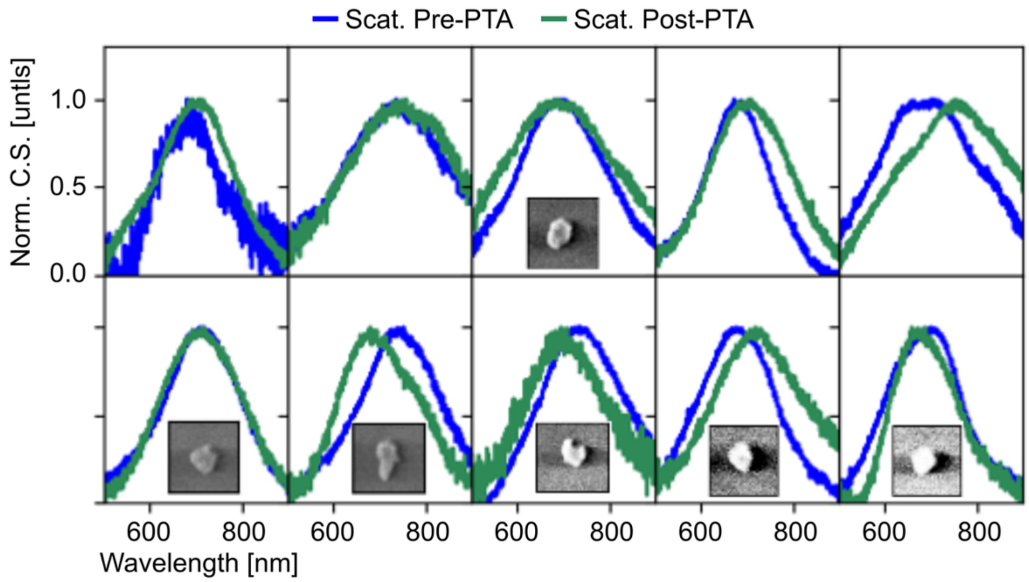

Figure S5. Long-term oxidation study of Mg NPs exposed to glycerol. NPs were inside the same glycerol filled cell (Fig. 2A) but did not undergo PTA measurements (no laser irradiation). Given that there is no global blueshift of the spectra, we conclude that no substantial glycerol-induced oxidation occurred over a time period of one week.

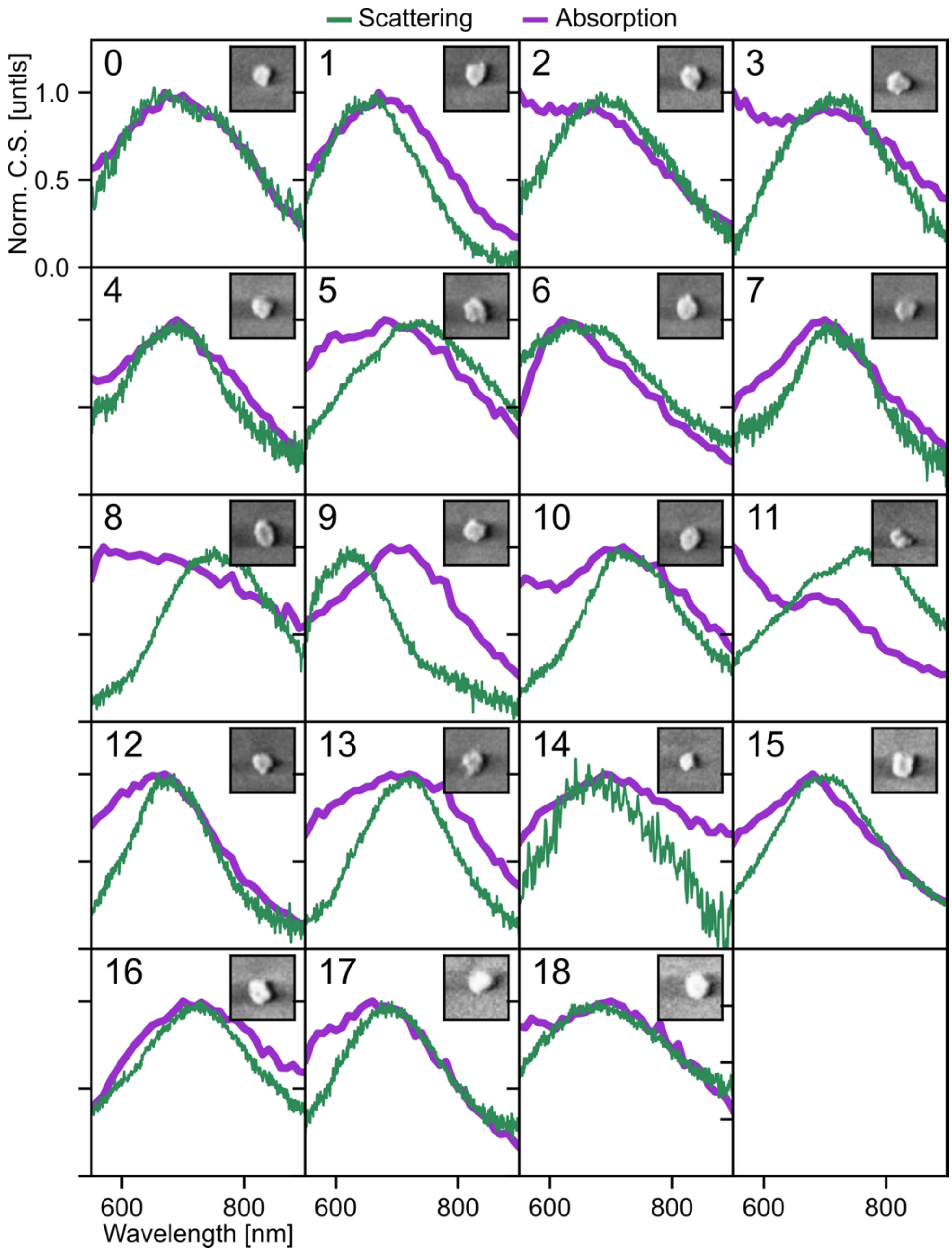

Figure S6. All 19 measured NPs with correlated SEM, normalized scattering (green), and normalized absorption (purple). NPs 1, 6, 7, and 9 are shown in Figure 2. SEM panel width is 490 nm.

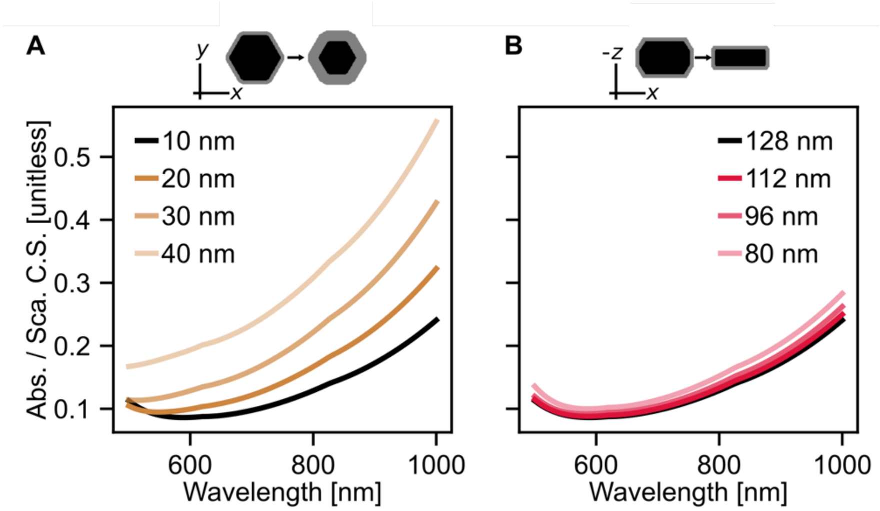

Figure S7. Absorption to scattering cross-section ratio of simulated Mg NP geometries from Figure 4C-D. (A) Increasing modelled oxidation increases absorption to scattering ratio, as expected because the metallic core becomes smaller and proportionally absorbs more. (B) Decreasing NP thickness slightly increases absorption to scattering ratio.

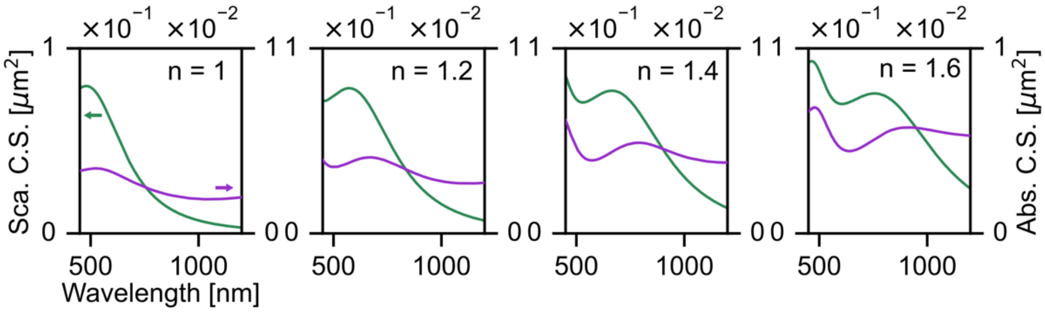

Figure S8. Mie theory with an increasing refractive index value for the infinite environment redshifts both absorption and scattering spectra, increases resonance offset, and broadens absorption. Mie theory including $\ell = 10$ multipoles of Mg nanospheres with a diameter of 170 nm. (Left to right) Increasing refractive index of the infinite environment from n = 1 (air), n = 1.2, n = 1.4, n = 1.6.

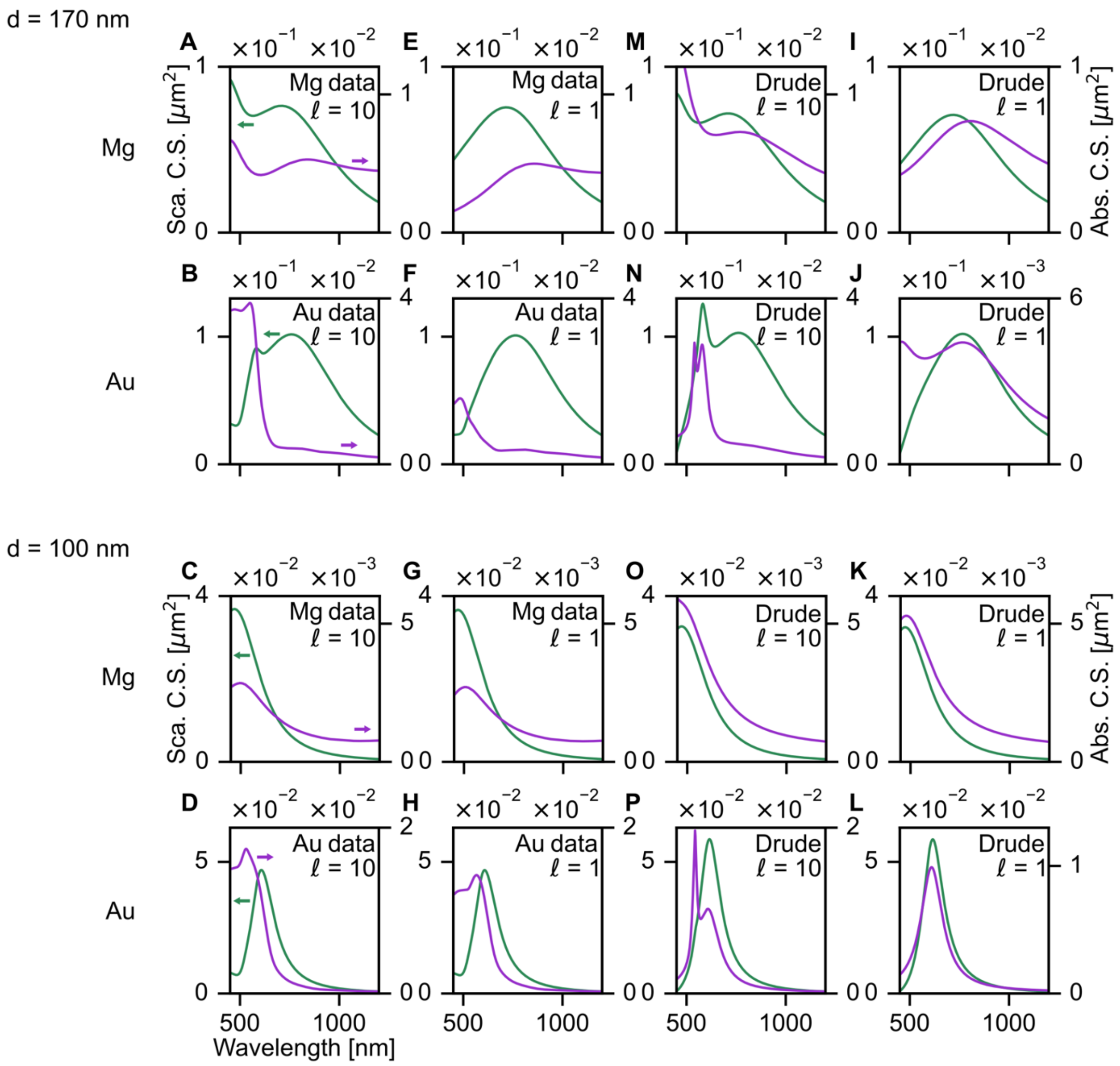

Figure S9. Mie theory reproduces trends from simulations of facetted spheroids and demonstrates that broadening and resonance shift persist with the simplest model: Drude dielectric function and ℓ=1 (dipole mode). Quantitative agreement between Mie theory and experiment is not intended herein; the purpose of this analysis is to understand trends in experiment through simulation. Au is included as a comparison to Mg. An average dielectric background is used to approximate the glass substrate and glycerol environment, n=1.5. (A-D) For Mie theory with the most accurate parameters (experimentally-obtained dielectric data,

including modes up to 10), absorption is broader than scattering for Mg, and for the larger Mg spheres (A), the absorption and scattering resonances are shifted. A comparison to Au (B,D) is inappropriate here as the lineshape is dominated by interband transitions and it is not possible to isolate the dipole resonance. (E-H) These trends persist for Mg, and it is still not possible to isolate the absorption resonance in Au due to interband transitions. (M-P) Removing interband transitions by using a Drude model for Mg and Au shows that the Mg trends are maintained and that the Au response is dominated by higher order modes. (I-L) With no interband transitions or higher order modes present, for a large Mg sphere (I), the shift and broadening are present, while for a Au sphere (J), the resonances occur at the same wavelength without significant broadening. Thus, the broadening and shift are due to the dielectric function of Mg impacting the ℓ=1 mode; the oxide shell present in Mg, the facetted nature of the Mg NP, higher order modes, or interband transitions only modulate this Mg intrinsic behavior. To determine the exact cause from the dielectric function, see Fig. S10.

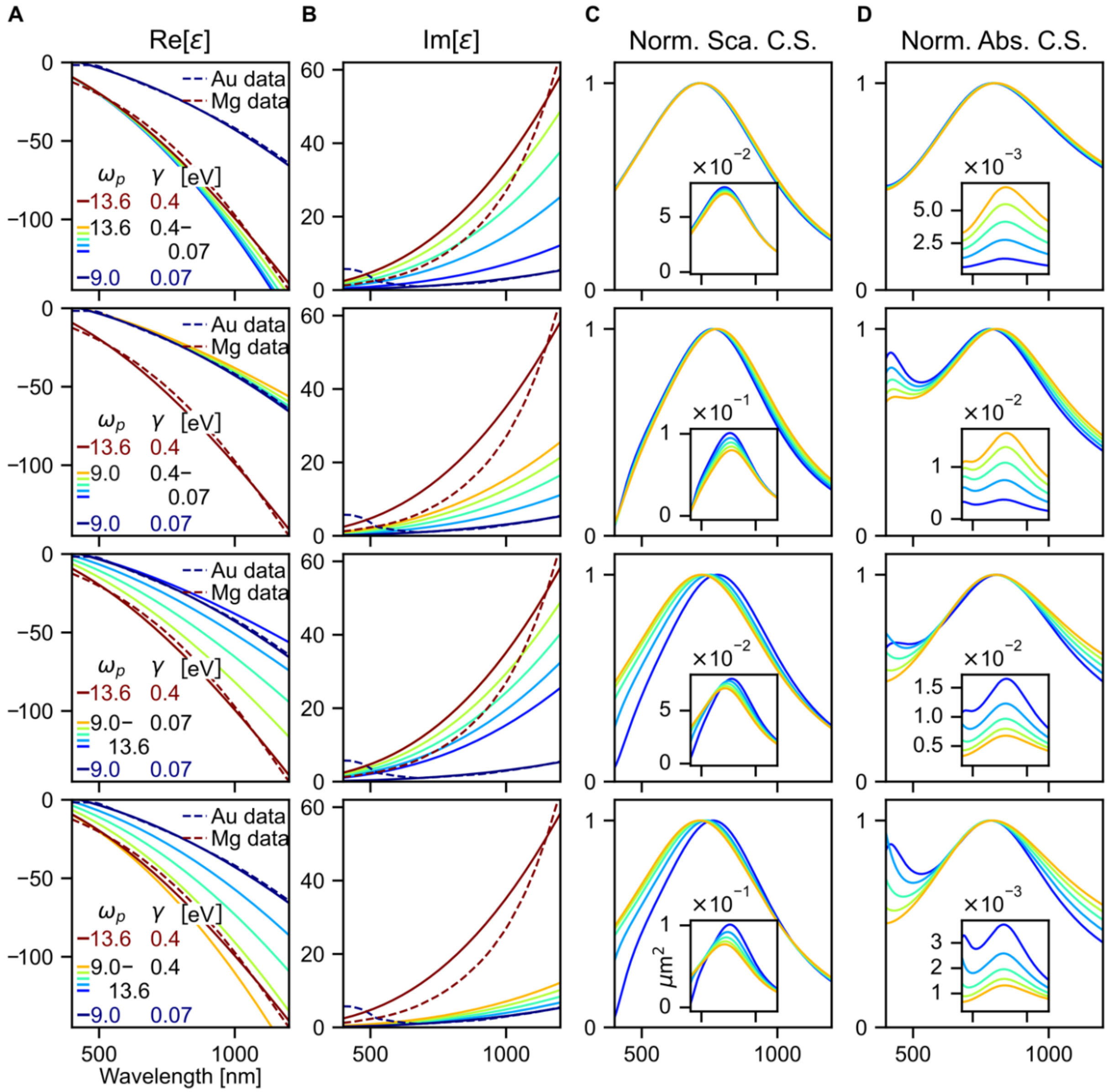

Figure S10. Interpolating plasma frequency and Drude damping from Au to Mg shows that the shift and broadening are due to the increased value of Re[$\epsilon(\omega)$] from an increased plasma frequency, ω_p. Calculations are done using Mie theory, in which the sphere diameter is 170 nm with only the ℓ = 1 contribution considered in an average dielectric environment, n = 1.5. (A-B) Real and imaginary dielectric function from experiment (dark blue dashed, Au Johnson & Christy data[7], maroon dashed, Mg Palik data[6]). Fits to data are plotted in corresponding colors

and solid line traces, with plasma frequency, ω_p, and Drude damping, γ, indicated in legend. ϵ_∞ is kept constant among all traces. Each row varies one parameter (γ in rows 1-2, ω_p in rows 3-4) while pinning the other at either the Au (rows 1,4) or Mg (rows 2,3) value. (C-D) Normalized scattering and absorption from the dielectric function indicated by colors corresponding to (A-B), with un-normalized spectra shown in insets. Increasing the Drude damping (rows 1-2) primarily increases Im[$\epsilon(\omega)$] and does not significantly shift or broaden the resonances. Increasing the plasma frequency increases Re[$\epsilon(\omega)$] and increases Im[$\epsilon(\omega)$] for small γ (row 3), and primarily only increases Re[$\epsilon(\omega)$] for larger γ (row 4). These changes result in blueshifting of the scattering spectrum and broadening of absorption. Therefore, the shift and broadening is from Mg's larger Re[$\epsilon(\omega)$], which originates from a larger ω_p.

REFERENCES

(1) Lomonosov, V.; Hopper, E. R.; Ringe, E. Seed-Mediated Synthesis of Monodisperse Plasmonic Magnesium Nanoparticles. *Chem. Commun.* **2023**, *59* (37), 5603–5606. https://doi.org/10.1039/d3cc00958k.

(2) Olson, J.; Dominguez-Medina, S.; Hoggard, A.; Wang, L.-Y.; Chang, W.-S.; Link, S. Optical Characterization of Single Plasmonic Nanoparticles. *Chem. Soc. Rev.* **2015**, *44* (1), 40–57. https://doi.org/10.1039/C4CS00131A.

(3) Yorulmaz, M.; Nizzero, S.; Hoggard, A.; Wang, L. Y.; Cai, Y. Y.; Su, M. N.; Chang, W. S.; Link, S. Single-Particle Absorption Spectroscopy by Photothermal Contrast. *Nano Lett.* **2015**, *15* (5), 3041–3047. https://doi.org/10.1021/nl504992h.

(4) Draine, B. T.; Flatau, P. J. Discrete-Dipole Approximation for Scattering Calculations. *J Opt Soc Am A* **1994**, *11* (4), 1491–1499. https://doi.org/10.1364/JOSAA.11.001491

(5) Boukouvala, C.; Ringe, E. Wulff-Based Approach to Modeling the Plasmonic Response of Single Crystal, Twinned, and Core-Shell Nanoparticles. *J Phys Chem C* **2019**, *123* (41), 25501–25508. https://doi.org/10.1021/acs.jpcc.9b07584.

(6) Palik, E. D. *Handbook of Optical Constants of Solid*; Academic Press, 1998.

(7) Johnson, P. B.; Christy, R. W. Optical Constants of the Noble Metals. *Phys. Rev. Lett.* **1972**, *11*, 4370–4379. https://doi.org/10.1103/PhysRevB.6.4370
